# Evidence of the role of Midkine and Pleiotrophin in the pathogenesis of Familial Mediterranean Fever

**DOI:** 10.1007/s00431-026-06981-9

**Published:** 2026-05-18

**Authors:** Zeinab Y. Abdallah, Ghada Nour Eldeen, Mona F. Sokkar, Rania Fawzy Mahmoud abdelkawy, Randa S. Lotfy, Shymaa H. Hussein, Khaled Hamed, Hala T. El-Bassyouni

**Affiliations:** 1 Human Genetics and Genome Research Institute, Biochemical Genetics Department, National Research Centre, Cairo, Egypt; 2 Human Genetics and Genome Research Institute, Molecular Genetics and Enzymology Department, National Research Centre, Cairo, Egypt; 3 Human Genetics and Genome Research Institute, Immunogenetics Department, National Research Centre, Cairo, Egypt; 4 Human Genetics and Genome Research Institute, Cytogenetics Department, National Research Centre, Cairo, Egypt; 5 Human Genetics and Genome Research Institute, Clinical Genetics Department, National Research Centre, Cairo, Egypt

**Keywords:** Familial Mediterranean Fever, Subclinical inflammation, MEFV gene, Midkine, Pleiotrophin

## Abstract

Familial Mediterranean Fever (FMF) is a chronic autoinflammatory illness that follows a pattern of periodic attacks; however, the majority of cases experience ongoing subclinical inflammation during the attack-free periods that could lead to the development of anemia, splenomegaly, heart disease, and the major complication, amyloidosis. Midkine/Pleiotrophin (MDK/PTN) are heparin-binding cytokines, with upregulated expression in various inflammatory and malignant diseases. Thus, the present research aims to identify the role of MDK and PTN in the pathogenesis of chronic inflammation and to explore the potential of both cytokines as new markers of subclinical inflammation in FMF. Blood samples from 30 FMF patients and 30 controls were collected to assess gene expression and protein levels of MDK and PTN by qRT-PCR and ELISA, respectively. Statistical correlation between different parameters and bioinformatic analysis to link the *MEFV* gene to the relevant genes was also performed. The expression of MDK and PTN genes was significantly higher in FMF children than in healthy subjects. Additionally, the serum level of MDK showed a fourfold increase in patients compared to controls (*p* < 0.001). The levels of PTN were also higher in cases, but without reaching the significance level (*p* = 0.096). MDK gene expression was inversely related to ESR level and positively correlated to serum level of MDK and anemia occurrence.

*Conclusion*: Collectively, our results disclosed that Midkine could be identified as a useful clinical tool in diagnosing and managing systemic and subclinical inflammation in FMF cases, while Pleiotrophin might provide additional information on acute inflammatory processes.

**What is Known:**

• *Familial Mediterranean Fever (FMF) is a genetic autoinflammatory disorder characterized by recurrent bouts of systemic and acute inflammation.*

• *Midkine and Pleiotrophin are heparin-binding growth factors often associated with inflammatory responses.*

**What is New:**

• *Midkine could be identified as a reliable clinical biomarker for both diagnosing and managing systemic and subclinical inflammatory phase in FMF patients.*

• *Pleiotrophin might provide additional information on acute inflammatory processes.*

## Introduction

Familial Mediterranean Fever (FMF) is a recessively inherited autoinflammatory disease that commonly affects Mediterranean people, mostly Turks, Jews, Armenians, and Arabs, with a prevalence varying from 1:500 to 1:1000 in endemic countries [1]. The typical symptoms of the disease include recurring episodes of fever, abdominal pain, and the inflammation of serosal membranes [2]. FMF is caused by variants in the Mediterranean fever gene (*MEFV*), also known as pyrin, that is predominantly expressed in neutrophils, playing a central role in regulating the innate immune response and the inflammatory processes [1]. The inflammatory episodes of the disease are mediated by a massive influx of neutrophils into the serous cavities with elevation in the levels of acute-phase proteins and inflammatory cytokines, that have been detected during the acute attacks [3]. However, subclinical inflammation continues during the attack-free period and could lead to the development of anemia, splenomegaly, heart disease, decreased bone mineral density, and the most fetal complication, amyloidosis, without the usual signs and symptoms of the disease. Hence, identifying the predictors of persistent inflammation is crucial and would help capture the patients’ predisposition to persistent inflammation [4]. Moreover, systemic inflammation in FMF requires regular monitoring and evaluation of patients for better management and prevention of FMF-related complications [4]. However, the clinical utility of novel inflammatory parameters has not been well explored in FMF [5], and new biomarkers are needed to monitor disease progression and treatment response [6].

Increasing evidences suggested the essential role of the heparin-binding growth factor, Midkine (MDK), along with the structurally related growth factor Pleiotrophin (PTN), belongs to the family of neurite outgrowth-promoting factors in acute and chronic inflammatory diseases [7]. MDK expression is driven by the master regulator of pro-inflammatory pathways, nuclear factor kappa of activated B cells (NF-kB), which can be activated by reactive oxygen species (ROS), pro-inflammatory cytokines, and bacterial components, indicating the relevance of MDK signaling in response to inflammatory stimuli [8]. Previous investigators have also revealed that MDK promotes inflammation via the induction of pro-inflammatory cytokines, such as IL-1β, the recruitment and activation of inflammatory immune cells, and the attenuation of regulatory mechanisms [8]. Additionally, the patients with neurodegenerative, ischemic diseases, autoimmune, and inflammatory disorders had upregulated levels of MDK, proposing its potential use as a disease marker [9]. The function of PTN in the inflammatory process has also been proven by stimulating the expression of mRNAs, leukocyte recruitment, and generation of the inflammatory cytokines in cells of the immune system [10]. Although PTN and MDK are expressed in many different inflammatory diseases, their function in that regard is not well elucidated.

Hence, the purpose of our investigation was to assess the serum concentrations and the relative gene expression of MDK and PTN in Egyptian FMF cases during the quiescent state to explore the role of both mediators in the pathogenesis of chronic inflammation of FMF. We further investigated the relations of MDK and PTN to clinical features, inflammatory markers, and *MEFV* genotypes to assess their potential as novel disease markers.

## Subjects and methods

### Ethical approval

The present study has been approved by the local Ethics Committee of the National Research Centre (NRC), Egypt, according to the principles expressed in the Declaration of Helsinki, 1975, with approval number 02431223. Written informed consent was provided by all participants and/or their parents prior to inclusion in this study.

### Subjects

The current investigation included 30 FMF children during the quiescent state of the disease and 30 healthy controls matched for age and sex. FMF cases were recruited from the Clinical Genetics clinic at the Medical Centre of Excellence, NRC. The diagnosis of FMF cases was established clinically according to the Tel-Hashomer criteria [11], and the disease severity was also assessed using the criteria of International Severity Scoring System for FMF (ISSF) [12].

A quiescent disease state was defined as the absence of all the signs and symptoms of the FMF attacks for at least two consecutive weeks. Patients having other systemic diseases (diabetes mellitus, chronic renal failure, malignancy, or ischemic heart disease), performing heavy exercises, or receiving medications other than Colchicine were excluded from the study.

## Methods

### Clinical examination

Patients were subjected to a detailed medical history, including demographic data, age, consanguinity, family history with three-generation pedigree construction, and clinical symptoms including fever, abdominal manifestations, chest manifestations, arthralgia, and erysipelas-like skin lesions. The frequency of attacks and doses of colchicine were also collected. Meticulous clinical evaluation and disease severity assessment using the scoring systems were also performed.

### Laboratory investigations

#### Biochemical analysis

Serum from patients and controls was obtained from clotted (15 min, room temperature) and centrifuged (15 min, 700 g) blood and stored at − 20 °C for subsequent assay. Serum Midkine and Pleiotrophin concentrations were evaluated using enzyme-linked immunosorbent assay (ELISA) kits (Human Midkine, Human Pleitropin, Innova Biotech Co., LTD., Beijing, China) according to the manufacturers’ instructions. Quantitative measurement of CRP, SAA protein, and vitamin D was also done using ELISA kits supplied by Elabscience Co. (USA), according to the manufacturer’s protocols. The erythrocyte sedimentation rate (ESR) was assessed manually by the Westergren method.

### Molecular studies

#### DNA extraction and MEFV mutation analysis

The DNA was isolated from peripheral blood samples taken on EDTA using the ZYMO Quick-DNA Miniprep Kit (Zymo, USA) according to the manufacturer’s guidelines. The DNA concentration was measured using a Nanodrop spectrophotometer (Thermo Scientific, Wilmington, USA). The genotyping of common 13 mutations of the *MEFV* gene (E148Q in exon 2, P369S and R408Q in exon 3, F479L in exon 5; M680I G/C, M680I G/A, M694V, M694I, K695R, V726A, A744S, R761H and I692 del in exon 10) was performed using real-time SNP genotyping commercial kits, followed by melting curve analysis (DNA Technology, Moscow, Russia) in accordance to the manufacturer’s protocol.

### RNA extraction

Total RNA was extracted from blood samples of patients and healthy subjects using direct-Zol Zymo RNA extraction kit (Zymo, USA) following the manufacturer’s guidance. RNA concentration and purity were measured using a Thermo Scientific Nanodrop spectrophotometer (Wilmington, USA). Isolated RNA was reverse transcribed to complementary DNA (cDNA) using the COSMO cDNA synthesis kit (COSMO, USA) according to the manufacturer’s protocols. Reverse transcription was performed under the following conditions: 5 min at 25 °C, 15 min at 45 °C and 5 min at 80 °C.

### Gene expression analysis using qRT-PCR

Real-time quantitative PCR (qRT-PCR) was carried out to quantify the expression levels of target genes (MDK and PTN) and the housekeeping gene (GAPDH) in the FMF cases and the controls using HERA SYBR green Master Mix (Cosmo, USA). Data was then collected and analyzed with the software accompanying the Light cycler 480 equipment (Roche, Germany) using a 2^− ΔΔCT^ method for calculating the relative quantification of the mRNA levels [13].

### Bioinformatics analysis

Analysis of functional association and interaction of the *MEFV* gene with our biomarkers and their associated genes was conducted using the GeneMANIA Cytoscape app [14]. Genes responsible for PTN and MDK protein production were examined in relation to the MEFV gene. Screening parameters for association links included protein and genetic interactions, pathways, co-expression, and protein domain similarity.

Variation’s influence on the final protein structure and stability was modeled using the Site-Directed Mutate webserver [15]. A three-dimensional structure for the wild-type protein was used from AF-O15553-F1-v6.pdb ((AlphaFold3 model v6 for human MEFV/pyrin, UniProt O15553)). All the identified mutations in our study (E148Q, P369S, M680I, M694I, M694V, V726A, and A744S) were evaluated.

### Statistical analysis

Analysis of data was performed using Statistical Package for the Social Sciences (SPSS) version 27. Description of the quantitative variables was presented in terms of means and standard deviations (SD). Description of qualitative variables was in terms of numbers (No.) and percent (%). The data was explored for normality using Shapiro–Wilk tests. Comparing different variables was done using both independent samples t-tests (assuming normality) and Mann–Whitney *U* tests (non-parametric alternative). Chi-square test (*χ*2) was used to detect the independence between groups of binary variables. Binary correlations were carried out using Pearson correlation. Receiver operating characteristic-area under the curve (ROC-AUC) analysis was done to examine the ability of PTN and MDK serum concentrations and gene expression values in differentiating FMF patients from controls. The level of significance for all tests was set at *p* ≤ 0.05.

## Results

### Demographic characteristics of FMF patients

Thirty FMF patients were enrolled. There was no statistically significant difference (*p* > 0.05) between the individuals with FMF and healthy controls according to age and gender status. The distribution in FMF cases included 16 males (53.3%) and 14 females (46.7%), with a mean age of 10.67 years. 33.3% of the patients were descended from consanguineous marriage, and 26.7% had a family history of FMF. The symptoms of abdominal pain (93.3%), fever (66.7%), arthritis (60%), and pleuritis (13.3%) were the predominant clinical symptoms. All the subjects were on colchicine (0.25–2 mg/day) treatment. The disease severity score was equally distributed into moderate and severe forms. The frequency of attack was 11.47 ± 2.9 episodes/year with an average attack duration of 131.07 ± 42.39 h, with broad individual variations. The laboratory assessment revealed anemia and vitamin D deficiency in 46.7% of the cases. The levels of the following markers were elevated: CRP, 29.57 ± 15.11 mg/L; ESR in the first hour, 28.53 ± 21.12 mm/h; ESR in the second hour, 67 ± 36.53 mm/h; and SAA, 110.03 ± 92.69 mg/L (Table 1).

**Table 1:** Population demography and clinical aspects of FMF cases

Parameters	FMF patients (%)
Demographic data	
Age (year)^#^	10.67 ± 5.19
Gender (male/female)^$^	16/14
Consanguinity^$^	10 (33.3)
Family history^$^	8 (26.7)
Clinical presentation^$^	
Fever	20 (66.7)
Abdominal pain	28 (93.3)
Arthritis	18 (60)
Pleuritis	4 (13.3)
Red rash skin	3 (10)
Severity^$^	
Moderate	15 (50)
Severe	15 (50)
Anemia	14 (46.7)
Deficient vitamin D (ng/mL)	14 (46.7)
FMF attacks^#^	
Frequency of attack/year	11.469 ± 2.9
Duration of attacks in hours/month	131.067 ± 42.39
Laboratory data^#^	
CRP (mg/L)	29.57 ± 15.11 (normal, < 5 mg/L)
ESR 1 st hour (mm/hour)	28.53 ± 21.12 (normal for children, ~ 0–10 mm/h)
ESR 2nd hour (mm/hour)	67 ± 36.53
Serum amyloid A (mg/L)	110.03 ± 62.69 (normal, < 10 mg/L)

*CRP* C-reactive protein, *ESR* erythrocyte sedimentation rate

^**#**^Data are represented as number (*N*) and percentage (%)

^$^Data are represented as mean ± SD

### MEFV genetic variants and related clinical features

**Fig. 1:**
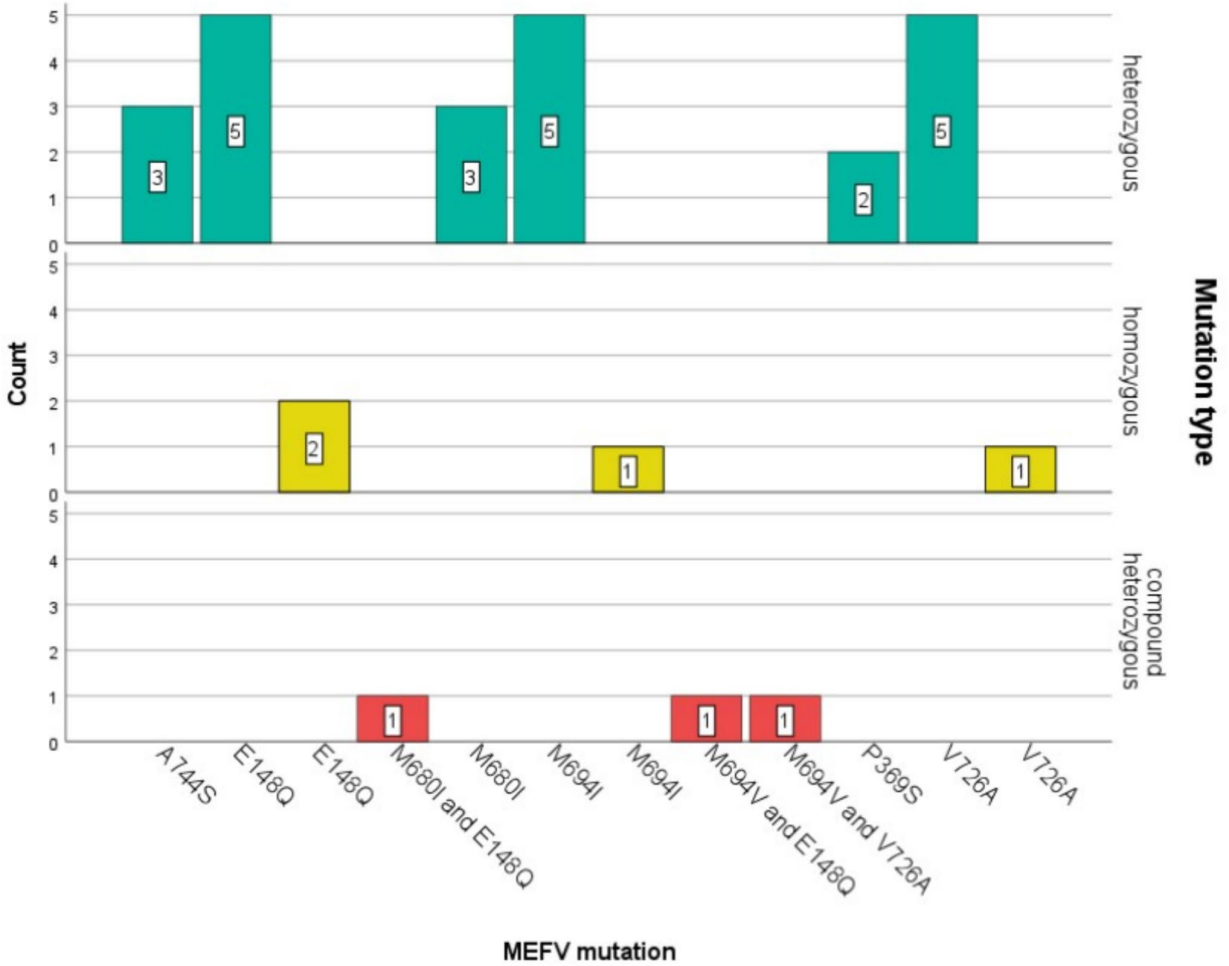
Bar graph demonstrating the distribution of the MEFV genetic variants among FMF patients. The heterozygous mutations were the most common, with E148Q, M694I, and V726A the most prevalent. Homozygous mutations were observed less frequently and usually involved the same mutations. Compound heterozygous mutations include M694I + E148Q and M694V + V726A, but the frequency was lower

*MEFV* genotype analysis in a cohort of 30 patients indicated that heterozygous mutations were predominant in 23 patients (76.7%), followed by homozygous mutations in four patients (13.3%) and compound heterozygous mutations in three patients (10%). Common variants were E148Q, M694I, and V726A within the heterozygous group, which represented 10–16.7% of the total, reflecting the prevalence of hotspots in Exons 2 and 10. The most common homozygous subtype accounted for 6.7%, and compound heterozygous variants were rare, at 3.3% each. This distribution indicates highly significant genetic heterogeneity in the FMF population, where heterozygous mutations are highly common (Fig. 1).

Most demographic, inflammatory, and biochemical parameters did not differ significantly among heterozygous, homozygous, and compound heterozygous FMF patients. However, mild trends toward higher CRP and serum amyloid A in heterozygous patients were observed. Moreover, attack frequency differed significantly (*p* = 0.028), being most prominent in homozygous patients. Colchicine dosage was marginally higher in homozygous patients (mean 1.5 ± 0.71 mg) compared to other groups, but these differences were not statistically significant (*p* = 0.372). Frequency of attacks varied significantly between groups (*p* = 0.028), with homozygous patients experiencing the highest mean frequency of attacks in the year (32.38 ± 10.31) while compound heterozygous and heterozygous patients showed lower and non-significantly different attack rates (Table 2).

**Table 2:** Phenotyping of genetic variants of FMF subjects

Variables	Heterozygous	Homozygous	Compound heterozygous	*p*-value
	Mean (SD)	Mean (SD)	Mean (SD)	
Age	10.04 ± 5.01	14.0 ± 6.16	11.0 ± 5.57	ANOVA = 0.383
CRP	31.61 ± 15.85	28.25 ± 8.77	15.67 ± 9.50	ANOVA = 0.230
ESR 1 st hour	31.39 ± 12.54	19.0 ± 12.08	19.33 ± 6.04	KW = 0.324
ESR 2nd hour	71.96 ± 37.52	43.25 ± 23.47	60.67 ± 39.63	ANOVA = 0.344
Serum amyloid A (mg/L)	121.13 ± 42.14	75.75 ± 33.77	70.67 ± 45.08	KW = 0.456
Frequency of attacks	9.22 ± 2.11^a^	32.38 ± 10.31^b^	0.86 ± 0.39^ab^	0.028*
Duration of attacks	129.13 ± 46.0	144.0 ± 41.86	128.67 ± 44.36	0.715
Colchicine	1.0 ± 0.538	1.5 ± 0.707	1.17 ± 0.764	KW = 0.372

*SD*, standard deviation; *KW*, Kruskal–Wallis *H* test

^a,b^Different small letters indicate statistical significance between the two groups

^*^Statistically significant *p*-value < 0.05

### Differential expression and circulatory levels of MDK and PTN in FMF patients

**Fig. 2:**
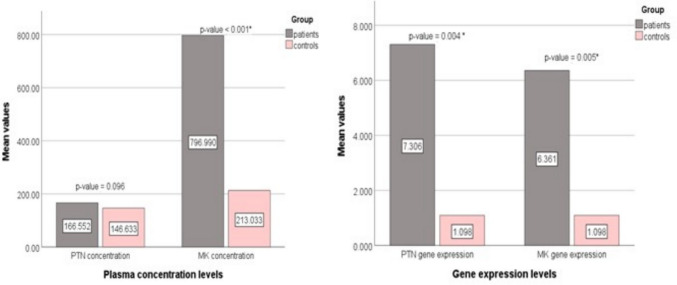
Bar charts for comparing midkine and Pleiotrophin serum concentrations and gene expression between patients and controls. *Statistically significant *p*-value < 0.05

MDK and PTN genes express significantly higher levels in FMF individuals than in healthy volunteers, with an average of 6.361 for MDK (*p* = 0.005) and an average of 7.306 for PTN (*p* = 0.004). This suggests that both genes were expressed following the inflammatory stimuli of FMF, thereby playing an active function in inflammatory and tissue remedial pathways. Meanwhile, MDK concentration was significantly higher in FMF cases at 796.99 pg/mL than in controls at 213.03 pg/mL, with a *p*-value of less than 0.001, proposing that MDK could serve as a strong biomarker related to inflammatory activity in FMF. In contrast, the levels of PTN were higher in patients (166.5 pg/mL) than in controls (146.63 pg/mL), but without reaching the significance value (*p* = 0.096) (Fig. 2).

### Correlation analysis of MDK and PTN expression profiles

Most demographic and clinical variables had no significant relation to MDK serum levels, except for anemia, where higher MDK levels reflect its association with hematologic stress (*R* = 0.386, *p* = 0.035). There is an inverse relationship between MDK and ESR (*R* =  − 0.441, *p* = 0.015), suggesting different inflammatory pathways. Taken together, a strong positive correlation between MDK gene expression and serum concentration (*R* = 0.898, *p* < 0.0001) speaks for the validity of MDK as an inflammatory mediator. Overall, MDK is self-consistent, increased in anemia, inversely related to chronic inflammation markers like ESR, and largely independent of most FMF clinical features (Table 3).

**Table 3:** Correlation of Midkine serum concentration and gene expression with demographic aspects in FMF participants

Variables	Serum Conc. correlation coefficient “*R*”	*p*-value	Gene Exp. correlation coefficient “*R*”	*p*-value
Age	0.228	0.226	0.138	0.469
Gender	− 0.301	0.106	− 0.286	0.126
Consanguinity	− 0.016	0.932	− 0.098	0.606
Family history	0.061	0.749	− 0.044	0.819
Fever	− 0.147	0.438	− 0.114	0.547
Abdominal pain	− 0.278	0.137	− 0.247	0.188
Arthritis	0.071	0.710	− 0.016	0.934
Pleuritis	− 0.147	0.437	− 0.102	0.592
Red rash skin	0.327	0.077	0.276	0.140
Severity	0.169	0.371	0.089	0.642
Anemia	0.386	0.035*	0.316	0.088
Deficient vitamin D	− 0.108	0.570	0.069	0.715
CRP (mg/L)	− 0.068	0.723	0.070	0.713
ESR 1 st hour	− 0.441	0.015*	− 0.364	0.048*
Serum amyloid A (mg/L)	− 0.138	0.465	− 0.226	0.230
Colchicine	0.275	0.141	0.203	0.283
Midkine gene exp	0.898	< 0.001*	-	-
Midkine serum conc	-	-	0.898	< 0.001*

^*^Statistically significant *p*-value < 0.05

Meanwhile, most demographic and clinical factors showed no significant correlation with PTN serum levels, except for PTN gene expression, which positively correlated (*R* = 0.437, *p* = 0.018*). This would mean that the higher the PTN mRNA expression, the higher the circulating PTN protein level, supporting its relevance in FMF subjects. PTN gene expression is positively correlated with fever episodes (*R* = 0.482, *p* = 0.007*), suggesting that PTN might be implicated in inflammatory responses. A near-significant trend with arthritis was observed (*R* =  − 0.350, *p* = 0.058). Other clinical factors presented weak correlations. Overall, PTN has been implicated as a biomarker for ongoing low-grade inflammation in FMF, associated especially with febrile episodes rather than with chronic manifestations, underlining its participation in inflammation and tissue remodeling processes during fever attacks (Table 4).

**Table 4:** Correlation of Pleiotrophin serum concentration and gene expression with demographic aspects in FMF subjects

Variables	Serum conc. correlation coefficient “*R*”	*p*-value	Gene expression correlation coefficient “*R*”	*p*-value
Age	− 0.194	0.314	− 0.135	0.476
Gender	− 0.191	0.320	0.023	0.903
Consanguinity	0.232	0.225	0.074	0.699
Family history	− 0.208	0.278	0.044	0.819
Fever	0.096	0.621	0.482	0.007*
Abdominal pain	− 0.139	0.473	0.077	0.685
Arthritis	− 0.047	0.809	− 0.350	0.058
Pleuritis	− 0.102	0.599	0.045	0.812
Red rash skin	0.027	0.889	− 0.250	0.182
Severity	− 0.091	0.639	− 0.270	0.150
Colchicine dose	− 0.107	0.579	− 0.120	0.528
Deficient vitamin D	0.029	0.881	0.141	0.458
CRP (mg/L)	− 0.185	0.337	0.158	0.405
Anemia	0.150	0.438	0.004	0.984
ESR 1 st hour	0.096	0.620	0.147	0.439
Serum amyloid A (mg/L)	0.197	0.305	− 0.094	0.620
Pleiotrophin gene exp	0.437	0.018*	-	-
Pleiotrophin serum conc	-	-	0.437	0.018*

*R*, Pearson correlation coefficient

^*^Statistically significant *p*-value < 0.05

### Differential expression of MDK and PTN across MEFV variant groups

MDK gene expression levels differed significantly between variant groups (*p* = 0.028). Post hoc comparisons suggested that compound heterozygous patients had the highest MDK expression, followed by homozygous patients, with heterozygous individuals showing the lowest expression. Notably, MDK serum concentration showed a tendency towards an increase related to the severity of the genotype, as it exhibited a trend toward higher levels in compound heterozygous patients (1589.13 ± 814.09 pg/mL) compared to homozygous and heterozygous groups, although it was not significant; thus, links between MDK pathways and genetic severity may be suggested. On the contrary, both serum concentrations and gene expression of PTN showed no significant variation between variant groups (*p* > 0.5). The mean PTN serum concentrations were slightly higher in heterozygous patients, but without statistical significance (Table 5).

**Table 5:** Comparable means of laboratory characteristics across different variant groups

Variables	Heterozygous	Homozygous	Compound heterozygous	*p*-value
	Mean (SD)	Mean (SD)	Mean (SD)	
Pleiotrophin serum conc	171.09 ± 53.43	146.67 ± 29.30	151.67 ± 35.47	KW = 0.592
Pleiotrophin gene exp	8.689 ± 3.952	3.996 ± 2.338	1.107 ± 0.412	KW = 0.394
Midkine serum conc	657.73 ± 511.44	1003.65 ± 426.56	1589.13 ± 814.09	KW = 0.075
Midkine gene exp	4.272 ± 2.947^a^	11.284 ± 10.642^ab^	15.819 ± 14.442^b^	0.028*

*SD*, standard deviation; *KW*, Kruskal–Wallis *H* test

^a,b^Different small letters indicate statistical significance between the two groups

^*^Statistically significant *p*-value < 0.05

**Fig. 3:**
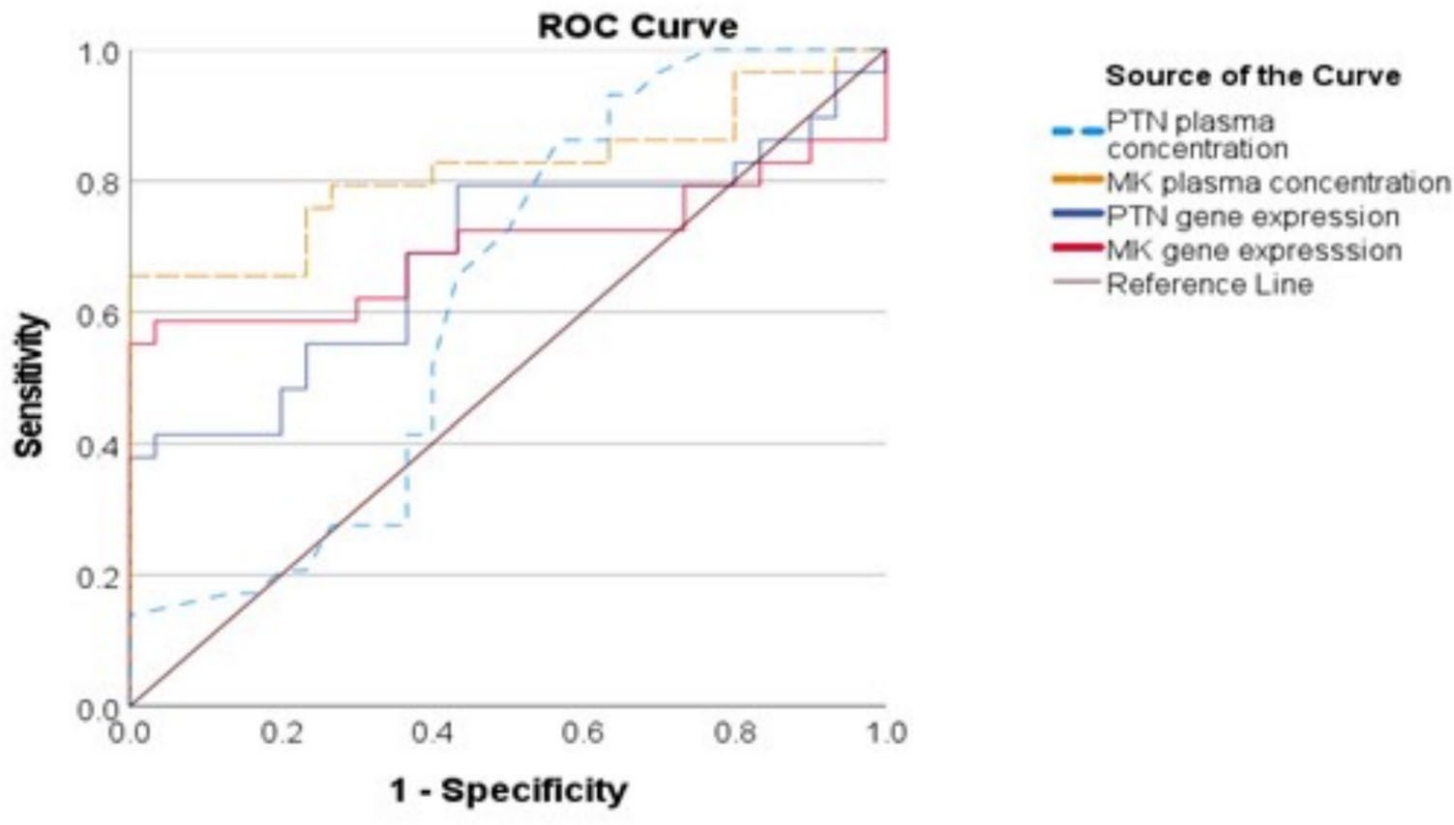
Receiver operating characteristic (ROC) curves for Midkine (MDK) and Pleiotrophin (PTN) assess their diagnostic performance in FMF participants versus controls. Four curves represent PTN plasma concentration, MDK plasma concentration, PTN gene expression, and MDK gene expression. The diagonal line indicates a non-discriminatory test (AUC = 0.5), and the curves displayed varying sensitivity and specificity of these biomarkers in differentiating FMF cases from healthy individuals

The ROC curve was conducted to evaluate the ability of PTN and MDK serum concentrations as well as gene expression values in distinguishing FMF from control subjects. The cutoff values for MDK and PTN concentrations were determined, with MDK showing a cutoff value of 384.5 (AUC, 0.816; confidence intervals (CI), 0.699–0.933; *p* < 0.001; sensitivity 65.5%; specificity 100%) and PTN a cutoff value of 123.5 (AUC, 0.626; CI, 0.480–0.773; *p* = 0.095; sensitivity 93.1%; specificity 63.3%). Meanwhile, for gene expression, MDK was 1.807 (AUC, 0.700; CI, 0.552–0.848; *p* = 0.008; sensitivity 65.5%; specificity 100%), and PTN’s cutoff value was 1.812 (AUC, 0.687; CI, 0.547–0.828; *p* = 0.013; sensitivity 65.2%; specificity 100%) (Fig. 3).

### Results of bioinformatics analysis

**Fig. 4:**
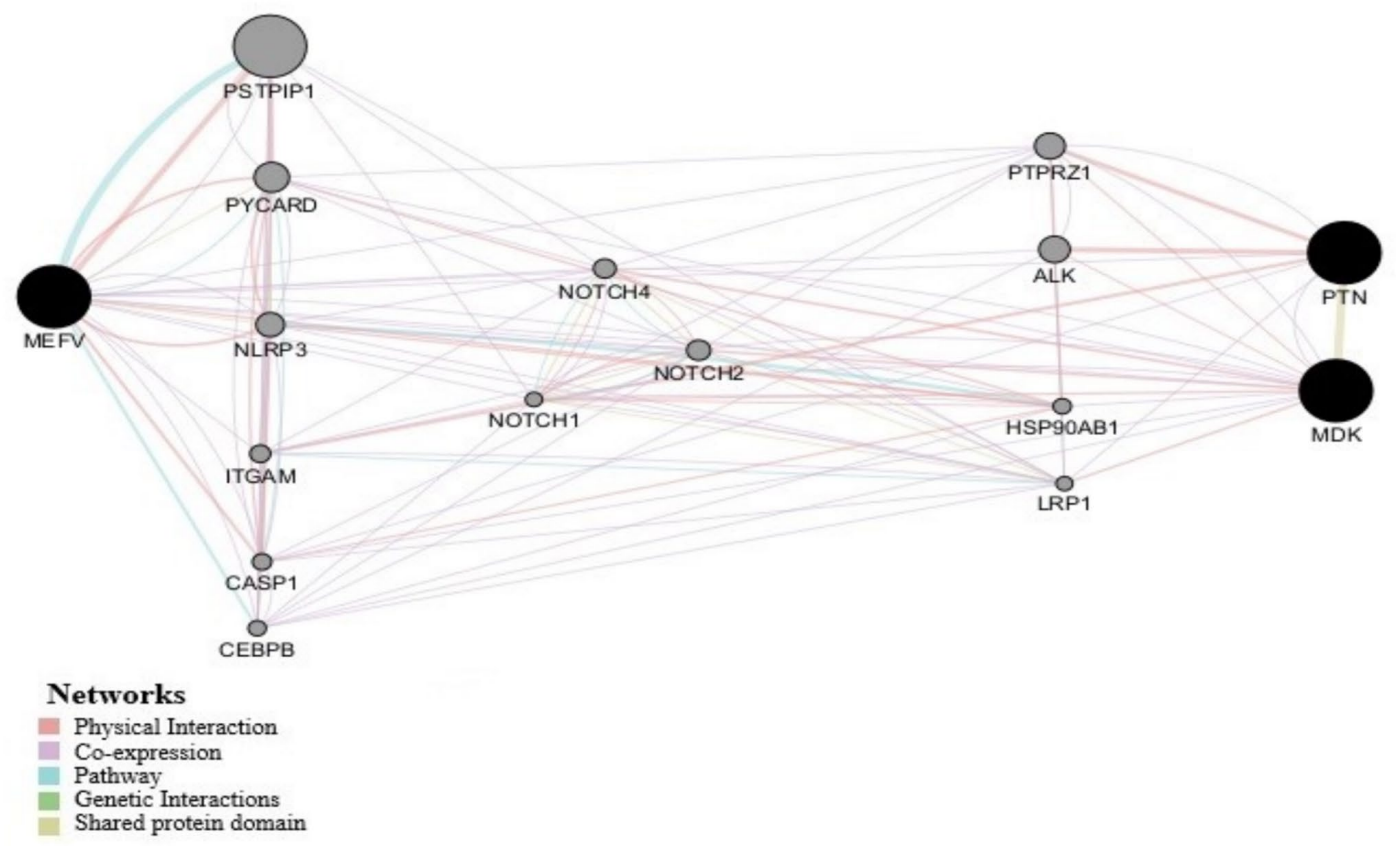
Interaction network analysis among the Mediterranean fever (*MEFV*) gene and genes associated with the production of MDK and PTN. Interaction analysis showed a set of intermediate genes including (*PSTPIP1*, *PYCARD*, *NLRP3*, *ITGAM*, *CASP1*, *CEBPB*, *NOTCH1*, *NOTCH2*, *NOTCH4*, *PTPRZ1*, *ALK*, *HSP90AB1*, *LRP1*) sharing co-expression, protein domain similarity, and pathway interaction links connecting all *MEFV*, *MDK*, and *PTN* genes

We studied the link between the *MEFV* gene and the relevant genes associated with the biomarkers under investigation to the best of our knowledge. Gene interaction network analysis using the GeneMANIA Cytoscape app revealed that the *MEFV* gene has both direct and indirect co-expression links with genes responsible for producing the biomarkers PTN and MDK (Fig. 4).

The Table 6 displayed the stability of the mutated protein structure. The analysis indicated that five mutations (E148Q, M680I, M694I, M694V and V726A) resulted in increased structural stability. Two mutations (P369S and A744S) caused reduced stability with predicted ΔΔGΔ*G* difference from the wild type of (− 0.63 and − 0.31, respectively). However, the most common mutation observed, M694I, led to increased residue solvent accessibility compared to the wild type. It also resulted in the loss of sidechain-sidechain hydrogen bond formation at the respective residue location suggesting a significant conformational change in the structure at the mutation site. This mutation caused a positive increase in ΔΔG by 0.91, resulting in a more stable structure than the wild type. The M680I mutation showed the slightest difference in ΔΔG among all studied mutations with only a 0.07 change due to the variation without any significant changes in protein structural properties. On the other hand, the P369S mutation caused a decrease in ΔΔG by − 0.63, leading to a less stable structure than the wild type (Table 6).

**Table 6:** Mutated protein structural stability analysis in FMF cases

Mutation	Residue	RSA (%)	Depth (Å)	OSP	SS	SN	SO	Predicted ΔΔ*G*	Outcome
E148Q	Wild type	97.6	3.2	0.04	False	False	False	0.11	Increased stability
	Mutation	108.4	3.2	0.04	False	False	False		
P369S	Wild type	96.6	3.1	0.09	False	False	False	− 0.63	Reduced stability
	Mutation	100.5	3.1	0.1	False	False	False		
M680I	Wild type	11.2	5.2	0.33	True	False	False	0.07	Slightly Increased stability
	Mutation	12.3	5.1	0.25	False	False	False		
M694I	Wild type	47.8	3.9	0.26	False	False	False	0.91	Increased stability
	Mutation	49.9	3.6	0.27	False	False	False		
M694V	Wild type	47.8	3.9	0.26	False	False	False	1.16	Increased stability
	Mutation	50.4	3.4	0.27	False	False	False		
V726A	Wild type	59.9	3.5	0.27	False	False	False	0.12	Increased stability
	Mutation	59.9	3.3	0.22	False	False	False		
A744S	Wild type	20.0	4.0	0.32	False	False	False	− 0.31	Reduced stability
	Mutation	16.9	4.2	0.33	True	True	False		

*OSP*, occluded surface packing; *RSA*, relative solvent accessibility; *SN*, sidechain-main chain amide hydrogen bond; *SO*, sidechain-main chain carbonyl hydrogen bond; *SS*, sidechain-sidechain hydrogen bond

## Discussion

Familial Mediterranean Fever (FMF), a prominent autoinflammatory disorder, is often induced by mutations within the *MEFV* gene. The gene codes for pyrin, which is mainly found in neutrophils and plays a pivotal role in the immune system. The pyrin protein is implicated in inflammasome formation and inflammatory mediator activation. Dysfunctional pyrin expression, noticed in FMF patients, leads to overproduction of interleukin-1 β (IL-1 β) and tumor necrosis factor α (TNF α), causing subclinical and inflammatory symptoms in different body parts [16].

Midkine is a multifaceted cytokine that plays a role in cell migration, growth, and survival. It is overexpressed in a number of inflammatory and autoimmune diseases, including sepsis and rheumatoid arthritis [8, 17]. Conversely, Pleiotrophin contributes to immunological and tissue repair. Its overall biomarker activity has not been fully studied, although it may show pro-inflammatory or repair actions depending on the biological setting [18, 19].

By assessing their gene expression and serum levels in Egyptian pediatric FMF patients, the current study aimed to explore the potential role of Midkine (MDK) and Pleiotrophin (PTN) cytokines in the pathophysiology of Familial Mediterranean Fever (FMF). Additionally, it investigated the relationships between MDK and PTN, clinical characteristics, inflammatory markers, and *MEFV* genotypes in order to evaluate their potential diagnostic utility as biomarkers of disease activity.

According to previously published data, 90% of cases of FMF manifest before the age of 20 [20], which was confirmed by the study as the mean age of 10.67 years for patients with FMF, indicating a childhood onset disease. Approximately one-third of cases had consanguinity, and more than 25% had a positive family history, underscoring the autosomal recessive inheritance pattern prevalent in North African and Middle Eastern populations [21].

Clinically, the most prevalent symptoms were fever, arthritis, and abdominal pain, and elevated inflammatory markers such as serum amyloid A (SAA) and erythrocyte sedimentation rate [22]. The rise in acute-phase reactants of our cohort is indicative of basal subclinical inflammation as opposed to acute disease activity. Furthermore, heterozygous *MEFV* variants were more common than homozygous and compound heterozygous mutations, according to genotypic analysis, which is in line with previously documented Mediterranean mutation patterns [23]. Genotype–phenotype relationships associated with severe conditions were highlighted by the association between homozygous mutations and more frequent disease attacks. However, there was no significant difference in inflammatory marker levels between genotypic groups; this could be due to individual variability, timing of sample collection, and colchicine treatment [22].

The current investigation found that MDK and PTN were markedly overexpressed at the gene expression level in FMF participants, suggesting that these growth factors may play a role in the inflammation linked to FMF. MDK is a potent pro-inflammatory mediator that is activated in autoimmune and inflammatory diseases, where it enhances cytokine signaling, neutrophil recruitment, and endothelial activation [8]. Notably, a nearly fourfold increase in circulating MDK was observed in FMF patients, indicating a strong correlation between MDK mRNA expression levels and MDK serum concentrations. This finding supports MDK’s validity as a systemic inflammatory marker, in line with earlier research in autoimmune/infectious diseases [24], since it has been reported that TNF-α and IL-1β contribute to raising the MDK expression significantly [25]. These results highlighted that MDK could be a part of how inflammasomes cause inflammation in FMF.

Interestingly, PTN mRNA was upregulated significantly, while PTN levels in the blood were not higher than normal control levels. This could support earlier findings that PTN mostly acts locally in tissues, with limited protein secretion into the blood [26]. Also, there is evidence that the protein can respond fast to an inflammatory trigger but is tightly controlled after transcription, so it is not easily found in serum [18].

More correlation analysis pointed out the different biological paths of MDK and PTN. MDK went hand in hand with anemia, which lines up with its known link to hypoxia-responsive pathways and blood stress [27]. Its negative link with ESR suggests MDK can measure short-term inflammation better than long-term inflammation, which was previously recorded in a study involving Crohn’s disease patients [28]. On the other hand, PTN levels were more tied to fever episodes and gene expression than to chronic inflammation, pointing to its role in inflammation and tissue changes during fever attacks [18].

Interestingly, MDK expression tended to be greater in compound heterozygote and homozygote MEFV genetic variants compared to controls which could be explained by the phenotypic variability, accompanied by diverse inflammatory cascades especially those associated with subclinical or tissue-level inflammation. Therefore, we thought that these findings indicated the emergence of midkine as an additional biomarker of non-classical inflammatory pathways, particular those related to tissue inflammation. In contrast, there was no change in PTN expression based on gene type. The receiver operating characteristic (ROC) curve showed that MDK can diagnose FMF well, with an area under the curve (AUC) of 0.816. Prior studies agreed that MDK has the ability to accurately identify inflammatory and autoimmune diseases [8]. Gene expression tests for MDK showed a lot of promise for diagnosis. This means a combined approach of gene expression testing and measuring relevant protein levels could help improve FMF diagnosis and monitoring. The PTN diagnostic test performed moderately and might serve as an extra indicator for low-grade inflammation between attacks, matching its known functions [18].

Furthermore, the bioinformatics analysis of the gene interaction network revealed a direct and indirect co-expression link between the *MEFV* gene and the genes responsible for producing MDK and PTN. Additionally, the evaluation of the protein structure stability of these biomarkers showed that the M694I, M680I, M694V, V726A, and E148Q mutations increased stability and enhanced protein function, contributing to the disease. In contrast, A744S and P369S variants suppressed it.

Considering all the previous observations, it has been possible to qualify MDK as a new, promising biomarker for FMF diagnosis and activity, while it seems that PTN reflects the transient inflammatory activity associated with recurrent febrile episodes. The increase in pleiotrophin gene expression during remission indicated that it might be involved in persistent low-level immune stimulation and tissue remodeling beyond overt clinical attacks. To the best of our knowledge, there are no studies evaluating the relative expression or the serum levels of MDK and/or PTN in cases having familial Mediterranean fever; that introduces a novelty for this paper and could be considered an advantage for future investigations on their joint application within the field of personalized medicine for FMF and other related autoinflammatory disorders.

## Conclusion

In summary, the results of this study provided new evidence associating the pro-inflammatory genetic pathway in Familial Mediterranean Fever with the involvement of the heparin-binding growth factors, Midkine (MDK) and Pleiotrophin (PTN). Our data confirmed that MDK is utilized as an accurate biological marker of subclinical inflammation in FMF cases, being significantly upregulated at both mRNA and protein levels in FMF subjects during remission phase, and is highly associated with, severity, and genetic predisposition. PTN, on the other hand, appears to be primarily involved as a locally acting and conditionally mediated biological agent and that was translated by increased gene expression as a persistent inflammatory activity between attacks with chronic tissue remodeling rather than chronic systemic inflammation. Crucially, as far as we are concerned, this study is the first one exploring the conjoint regulation at both gene expression and serum concentration levels in FMF cases, thus emphasizing primarily the novelty of the above results. Finally, based on this study’s data, MDK may be identified as an extremely useful clinical tool in diagnosing and managing systemic and acute inflammation in patients with FMF, while PTN might be identified as providing additional information on possible tissue sustained inflammatory processes between attacks.

## Data Availability

All data generated or analyzed during the study is included in the manuscript.
